# Dorso-ventral osteophytes of interphalangeal joints correlate with cartilage damage and synovial inflammation in hand osteoarthritis: a histological/radiographical study

**DOI:** 10.1186/s13075-022-02911-w

**Published:** 2022-09-29

**Authors:** Ilse-Gerlinde Sunk, Love Amoyo-Minar, Birgit Niederreiter, Afschin Soleiman, Franz Kainberger, Josef S. Smolen, Daniel Aletaha, Klaus Bobacz

**Affiliations:** 1 Department of Internal Medicine III, Division of Rheumatology, Medical University of Vienna, Waehringer Guertel 18-20, 1090 Vienna, Austria; 2 Department of Pathology, Medical University of Vienna, Vienna, Austria; 3 Department of Radiology and Osteology, Medical University of Vienna, Vienna, Austria

## Abstract

**Objective:**

To detect dorsally located osteophytes (OP) on lateral x-ray views and to correlate their presence with the extent of structural joint damage, determined by histologic grading (cartilage damage and synovial inflammation) and radiographic scoring in hand osteoarthritis (HOA).

**Methods:**

Distal interphalangeal (DIP) and proximal interphalangeal (PIP) joints were obtained from post mortem specimens (*n* = 40). Multiplanar plain x-rays were taken (dorso/palmar (dp) and lateral views). Radiographic OA was determined by the Kellgren and Lawrence classification. Joint samples were prepared for histological analysis and cartilage damage was graded according to the Mankin scoring system. Inflammatory changes of the synovial membrane were scored using the general synovitis score (GSS). Spearman’s correlation was applied to examine the relationship between histological and radiographical changes. Differences between groups were determined by Mann-Whitney test.

**Results:**

Bony proliferations that were only detectable on lateral views but reminiscent of OPs on dp images were termed dorso-ventral osteophytes (dvOPs). All joints displaying dvOPs were classified as OA and the presence of dvOPs in DIP and PIP joints correlated with the extent of histological and radiographic joint damage, as well as with patient age. Joint damage in osteoarthritic DIP and PIP joints without any dvOPs was less severe compared to joints with dvOPs. Synovial inflammation was mainly present in joints displaying dvOPs and correlated with joint damage.

**Conclusion:**

dvOPs are associated with increasing structural alterations in DIP and PIP joints and can be seen as markers of advanced joint damage. Detecting dvOPs can facilitate the diagnosis process and improve damage estimation in HOA.

## Key messages

- Dorso-ventral osteophytes (dvOPs) are radiographic signs of advanced joint alterations including synovial inflammation in interphalangeal osteoarthritis.
- dvOPs are highly specific and very sensitive particularly in distal interphalangeal joints.
- The evaluation of dvOPs as markers of joint damage might be implemented in radiographic scoring systems in the future.

## Introduction

Diagnosis of musculoskeletal disorders is often supported by clinical imaging. Despite emerging imaging options, such as musculoskeletal ultrasound [1], plain radiography is the perhaps most commonly used modality due to its high spatial resolution and wide availability. Thus, in hand osteoarthritis (HOA), the most prevalent joint disorder [2–4], plain radiography still represents the gold standard in imaging [5, 6] and is recommended by international societies [7, 8]. However, dorso/palmar (dp) images alone are suggested in the diagnostic process of HOA [7, 8]. This, in turn, is in conflict with the basic concepts of radiology that at least two projections are required to evaluate any structure [9].

In fact, dp, oblique and lateral views can be applied for the radiographic evaluation of the hand skeleton and multiplanar views are regarded important for routine imaging of the hands [10, 11]. Nevertheless, concerning HOA the question arises which structures would be importantly enhanced or exclusively displayed on oblique or lateral views compared with the dp view. This question was already partly addressed by investigating oblique radiographic views in patients with HOA [12]. The most frequent structural changes seen in oblique views that were not detectable by dp views were dorsally and/or ventrally located osteophytes (OP). The presence of these structures was associated with increased radiographic joint damage.

However, in this previous study, the associations were compiled purely from radiographic assessments and not at the tissue level. Here, we evaluated the presence of dorsally and/or ventrally located OPs with respect to histologically assessed structural cartilage damage and synovial inflammation. Moreover, we added another variable, namely joints without OA changes reflecting a control group.

## Patients and methods

### Joint specimens

Eighty interphalangeal joints—40 distal interphalangeal (DIP) and 40 proximal interphalangeal (PIP) joints—from 40 consecutive individual post mortem joint specimens (18 female and 22 male) were obtained at the Department of Pathology, Medical University of Vienna. Patients’ ages ranged from 33 to 96 years (median 66 years). Patients with a documented history of inflammatory joint disease, such as rheumatoid arthritis or psoriatic arthritis, were excluded. This cohort comprises the same specimens that were previously evaluated for histopathological analyses in HOA [13].

To obtain these specimens, the skin and subcutaneous tissues were carefully dissected until the DIP and PIP joints as well as the phalangeal bones became visible. Then, the first and third phalangeal bones were cut above the DIP joint and below the PIP respectively, so that the PIP and DIP joints could be obtained *in toto*. Of these 80 joints, 76 (37 DIP and 39 PIP joints) could be processed for histological and radiographic investigations.

Prior to dissection, both hands were clinically examined for Heberden and Bouchard nodes (palpation for firm/hard posterolateral rounded swelling and/or joined dorsal bars). Heberden and Bouchard nodes were classified as present or absent. If bony swelling was present, we selected the finger that clinically displayed the worst changes either on the left or on the right hand. If no nodes were present, the right hand was always used in accordance with the higher prevalence of OA on this side [14], and a computer program was employed to randomize which finger to dissect. The joint assessment was performed by an experienced rheumatologist (KB). This study was approved by the ethics committee of the Medical University of Vienna (No.: 409/2005).

### Radiographic and histological analysis

Plain radiography of the interphalangeal joints (dp and lateral views) was performed using a Philips Optimus 80 X-ray generator. A blinded assessment of the dp images was carried out according to the Kellgren and Lawrence (K/L) scoring system [15] by an experienced musculoskeletal radiologist (FK). Lateral views were evaluated for dorsally and/or ventrally based OPs, defined as OPs that are not apparent in the dp views. Lateral view images were assessed by consensus opinion of two experienced readers (IGS and KB) scoring together to obtain one score [16] (presence or absence of dorsal and/or ventral OPs). The whole set of lateral view images was read twice (4 weeks apart); reproducibility was very good with intraclass correlation coefficients (ICCs) for both DIP and PIP of 1.0.

Entire finger joints were prepared for histological analysis and stained with safranin-O/fast green and toluidine blue as previously described [13].

The modified Mankin score [17] was applied to grade structural damage of each sample histomorphologically. This scoring system is composed of four categories: cartilage structure (0–6 points), cartilage cells (0–3 points), staining (0–4 points), and tidemark integrity (0–1 point). Scores of each category are summed up to a total score with a possible maximum of 14 points. The samples displayed total Mankin scores ranging from 2 to 14 for DIP joints and 0 to 14 for PIP joints. A histopathological cut-off that distinguishes normal from OA cartilage was defined as a Mankin score > 5 [18]. As the Mankin score mainly focuses on the integrity of articular cartilage, it does not comprise bony alterations such as OPs. However, it is significantly correlated with the presence and extent of radiographic OPs and subchondral sclerosis as depicted elsewhere [13].

In order to evaluate the extent of inflammatory changes within the synovium, the histopathological general synovitis score (GSS) [19] was applied. Three components (lining layer hyperplasia, activation of resident cells and inflammatory infiltrate) were graded semi-quantitatively from 0 to 3 with a total score ranging from 0 to 9 (0 or 1, no synovitis; 2–4, low-grade synovitis; 5–9, high-grade synovitis) [19, 20].

### Statistics

To examine the relationship between dorsally and/or ventrally based OPs and the extent of joint damage, either reflected by the presence of histological (Mankin score, GSS) or radiographic changes (K/L score), the Spearman’s rank order correlation was used and expressed as *r*-values (*r*_s_). Mann-Whitney test was used to assess differences between groups. To evaluate intra-reader reliability, the intraclass correlation coefficients (ICC) were estimated. A *p*-value less than 0.05 was considered significant. Analysis was performed using MS Excel 2007 (Microsoft Corporation, Redmond, USA) and Prism 5 for Windows (GraphPad Software Inc., San Diego, USA).

## Results

### Dorso-ventral osteophytes

**Fig. 1:**
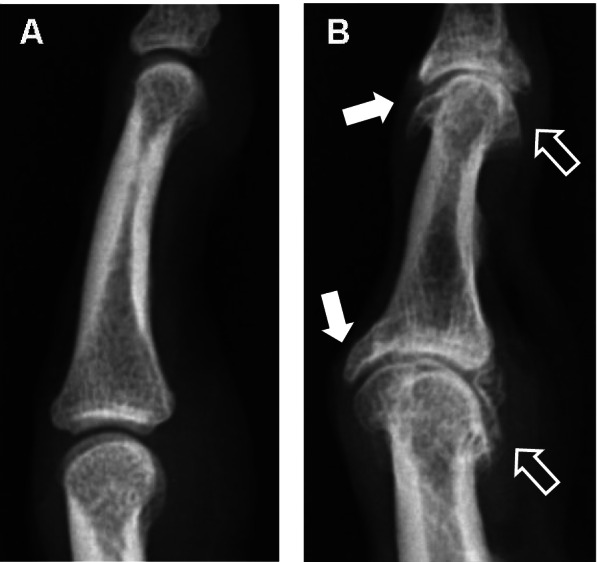
Example of lateral radiographic views of distal interphalangeal (DIP) and proximal interphalangeal (PIP) joints. **A** Normal DIP and PIP joints without dorso-ventral osteophytes (dvOPs). **B** DIP and PIP joints displaying dvOPs. The white arrows indicate dorsal osteophytes (OP), the outlined arrows mark ventral OPs

Dorsal and/or ventral OPs were defined as bony proliferations (spurs) that are only visible on lateral view images on the dorsal and/or ventral margins of OA joints, emerging either from the articular head, from the socket, or from both structures. These OPs form a convexly curved shape, growing proximally (Fig. 1).

Dorsal and ventral OPs were seen in 48.7% (*n* = 18) of evaluated DIP and less frequently in PIP joints (15.4%; *n* = 6). In the majority of the joints dorsal and ventral OPs occurred together (66.6%) and since joints with dorsal and/or ventral OPs did not show any difference in the extent of radiographic or histological joint damage, we summarized dorsal and ventral OPs under the term dorso-ventral osteophytes (dvOP). The percentage and distribution of dvOP compared with classic radiographic changes in HOA is shown in Table 1.

**Table 1:** Percentage and distribution of dorso-ventral osteophytes (dvOP) as well as classic radiographic changes in hand osteoarthritis. OP, osteophyte; JSN, joint space narrowing; DIP, distal interphalangeal joint; PIP, proximal interphalangeal joint. *p* values are provided comparing individual radiographic features between DIP and PIP joints. A *p* value <0.05 was considered significant. ns, not significant

	DIP joints	PIP joints	***p*** value
**dvOP**	48.7%	15.4%	< 0.003
**OPs**	56.8%	28.2%	< 0.02
**JSN**	67.6%	35.9%	< 0.007
**Subchondral sclerosis**	40.5%	33.3%	ns
**Subchondral cysts**	21.6%	12.8%	ns
**Erosions**	13.5%	10.3%	ns
**Malalignment**	2.7%	2.6%	ns

### Relationship of dorso-ventral osteophytes with histological and radiographic joint damage

**Fig. 2:**
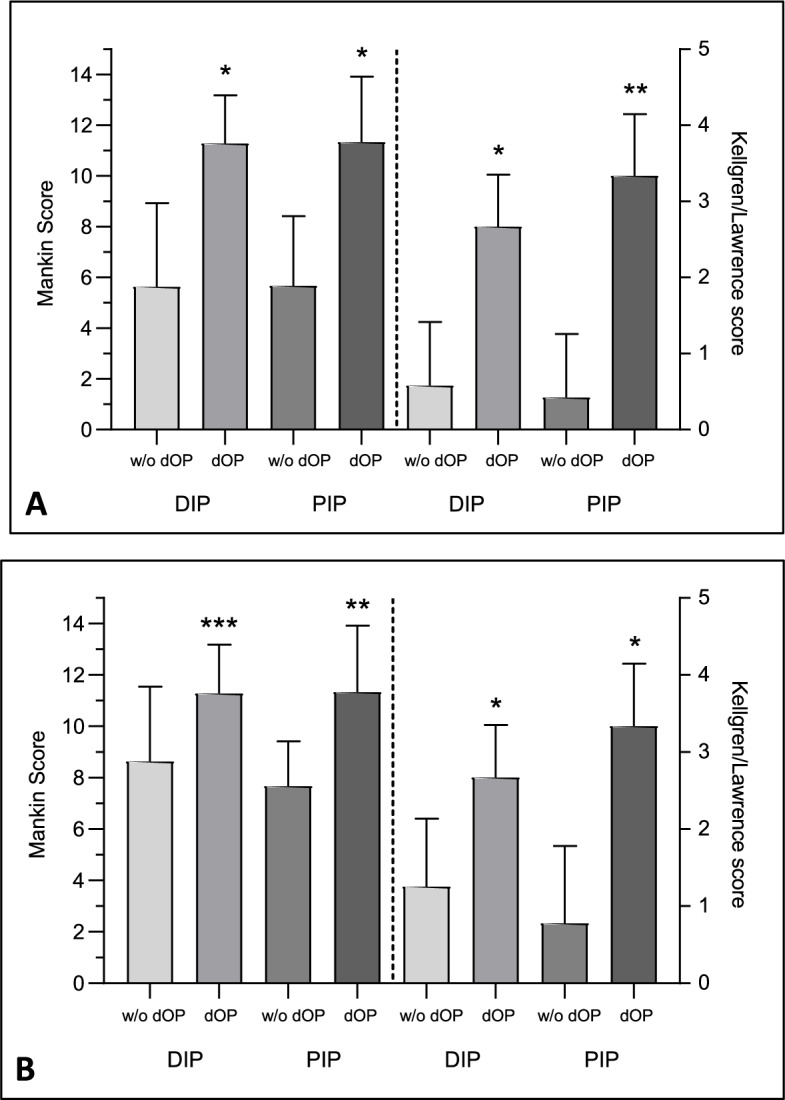
Joint damage in distal interphalangeal (DIP) and proximal interphalangeal (PIP) joints with or without the presence of dorso-ventral osteophytes (dvOPs). dvOPs were evaluated radiographically. Joint damage was determined by applying either a radiographic (Kellgren/Lawrence) shown on the right hand side of the graphs or histological (Mankin) score displayed on the left hand side of the graphs. The left and right *y*-axes show the distinct scales of the respective scores. **A** Data of the total cohort (joints with and without osteoarthritis) is represented. *p < 0.0001, **p < 0.005. **B** Data of a subgroup analysis of solely osteoarthritis joints (determined by a Mankin score > 5). *p < 0.001, **p < 0.01, ***p < 0.03. Error bars represent the standard deviations

All joints displaying dvOPs exhibited radiographic OA according to the K/L scale and reached > 5 points on the Mankin scale throughout. There was a direct association between the occurrence of dvOPs and the extent of structural joint damage, both on the histopathological (DIP: *r*_s_: 0.7; *p*<0.0001 / PIP: *r*_s_: 0.54; *p*<0.0005) and on the radiographic level (DIP: *r*_s_: 0.82; *p* < 0.0001/PIP: *r*_s_: 0.7; *p* < 0.0001). Concerning histopathological changes, in those DIP joints that displayed dvOPs the Mankin score, as surrogate marker of cartilage destruction, ranged from 8 to 14 (mean ± SD: 11.3 ± 1.9), while in DIP joints without dvOP, the mean Mankin score (mean ± SD) amounted to 5.6 ± 3.3; range: 2 to 14 (Fig. 2A). The difference between both groups was highly significant (*p* < 0.0001). In line with the histological alterations, the radiographic damage was significantly higher in DIP joints with dvOPs (K/L score: 2.7 ± 0.7) than in those without dvOPs (K/L score: 0.6 ± 0.8; *p* < 0.0001), as shown in Fig. 2A.

Regarding PIP joints, in the presence of dvOPs, we also recorded markedly higher joint damage on the histological (Mankin score in PIP joints with dvOPs: 11.3 ± 2.6 vs PIP joints without dvOPs: 5.7 ± 2.8; *p* < 0.0001), as well as on the radiographical level (K/L score in PIP joints with dOPs: 3.3 ± 0.8 vs PIP joints without dvOPs: 0.4 ± 0.8; *p* < 0.004) (Fig. 2A).

In order to evaluate the performance of radiographically detectable dvOPs, whose presence could be a sign of HOA, a diagnostic test analysis (sensitivity and specificity, positive predictive value and negative predictive value) was done (Table 2).

**Table 2:** Performance of radiographically detectable dvOPs with regard to diagnostic testing of HOA. HOA was determined by either radiographic (Kellgren/Lawrence) or histological (Mankin) scoring systems. PPV, positive predictive value; NPV, negative predictive value

*n*	OA according to Kellgren/Lawrence	OA according to Mankin
**dvOPs in DIP**		
**Sensitivity**	81.82% (59.72 to 94.81%)	69.23% (48.21 to 85.67%)
**Specificity**	100% (78.20 to 100%)	100% (71.51 to 100.00%)
**PPV**	100%	100%
**NPV**	78.95% (60.71 to 90.10%)	57.89% (43.58 to 70.99%)
**dvOPs in PIP**		
**Sensitivity**	54.55 % (23.38 to 83.25%)	25.00% (9.77 to 46.71%)
**Specificity**	100% (87.66 to 100%)	100% (78.20 to 100%)
**PPV**	100%	100%
**NPV**	84.85% (74.56 to 91.45%)	45.45% (39.81 to 51.22%)

### Osteoarthritic joints with and without dorso-ventral osteophytes and the extent of structural damage

In a subgroup analysis, we focused on those joints that were histologically graded as osteoarthritic (per definition Mankin score > 5). Our calculations revealed that out of all OA-DIP joints, 69.2% displayed dvOPs, while 30.8% showed none. In the latter group, the extent of structural damage was significantly lower (Mankin score: 8.6 ± 2.9) compared to OA-DIP joints with dvOPs (Mankin score: 11.3 ± 1.9; *p* < 0.03), as shown in Fig. 2B. Also, the radiographic damage (K/L score) was less severe in OA-DIP joints without dvOPs: 1.3 ± 0.9 vs OA-DIP joints with dvOPs: 2.7 ± 0.7; *p* < 0.0009 (Fig. 2B).

With regard to OA-PIP joints, dvOPs were present in 25%. The mean Mankin score in OA-PIP joints without dvOPs accounted for 7.1 ± 0.7 vs 11.3 ± 2.6 in OA-PIP joints with dvOPs and this difference was also significant (*p* < 0.004). In parallel, radiographic alterations in PIP joints without dvOPs were less severe compared to PIP joints with dvOPs (0.8 ± 1 vs 3.3 ± 0.8; *p* < 0.0002) (Fig. 2B).

### Relationship of dorso-ventral osteophytes with inflammatory changes

The GSS was applied to assess inflammatory affection of the synovial tissue. No synovitis (sum score 0 or 1) was found in 59.5% of DIP and 66.7% of the PIP joints, while low-grade synovitis (sum score 2–4) could be detected in 40.5% of DIP and 33.3% of PIP joints. No interphalangeal joint displayed high-grade synovitis. Table 3 shows the detailed distribution of synovial changes with regard to the GSS features.

**Table 3:** General synovitis score (GSS) in distal interphalangeal (DIP) and proximal interphalangeal (PIP) joints. The GSS consists of three features, lining layer hyperplasia, activation of resident cells, and inflammatory infiltrate that are graded from 0 to 3. The percentage of each graded feature in DIP and PIP joints is provided

	DIP joints				PIP joints			
GSS points	0	1	2	3	0	1	2	3
Synovial lining	18.9%	78.4%	2.7%	0	30.8%	69.2%	0	0
Synovial stroma	56.8%	37.8%	5.4%	0	61.5%	33.4%	5.1%	0
Inflammatory infiltrate	0	0	0	0	0	0	0	0

The GSS correlated well with histological and radiographic joint damage for both DIP (GSS vs Mankin score: *r*_s_ = 0.6; *p* < 0.0001; GSS vs K/L score: *r*_s_ = 0.77; *p* < 0.0001) and PIP joints (GSS vs Mankin score: *r*_s_ = 0.84; *p* < 0.0001; GSS vs K/L score: *r*_s_ = 0.79; *p* < 0.0001). Regarding dvOPs, we found an association between the presence of dvOPs and the GSS in DIP (*r*_s_ = 0.59; *p* < 0.0001) and PIP joints (*r*_s_ = 0.52; *p* < 0.0006).

**Fig. 3:**
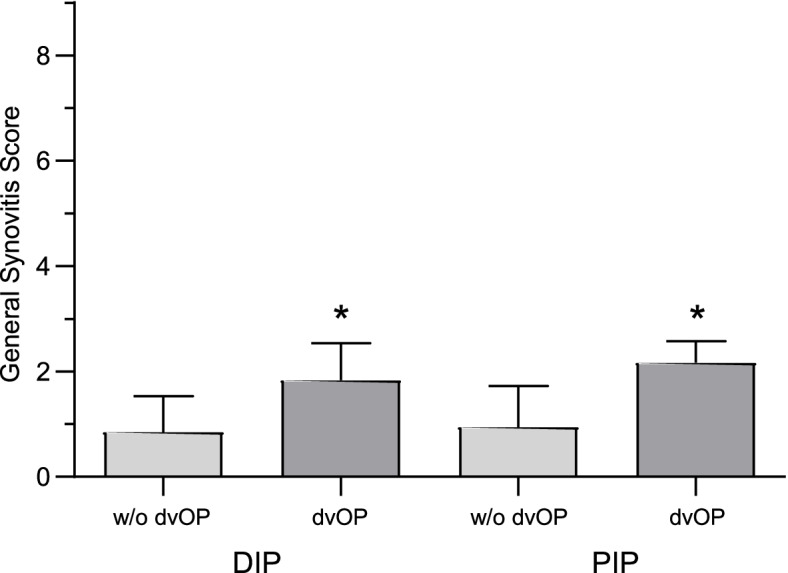
Inflammatory affection of synovial tissue in distal interphalangeal (DIP) and proximal interphalangeal (PIP) joints was assessed using the histopathological general synovitis score (GSS). Light gray columns represent joints without dorso-ventral osteophytes (dvOPs), while dark gray columns represent joints with dvOPs. *p < 0.0002

In those joints displaying dvOPs, the GSS was markedly increased compared to interphalangeal joints without dvOPs and this difference was significant for both DIP (mean GSS ± SD: joints with dvOPs: 1.83 ± 0.7 vs joints without dvOPs: 0.84 ± 0.67; *p* < 0.0002) and PIP joints (joints with dvOPs: 2.17 ± 0.41 vs joints without dvOPs: 0.94 ± 0.79; *p* < 0.0004), as shown in Fig. 3. The subgroup analysis of OA joints revealed a significant increase in the GSS score for DIP (*p* < 0.03) and PIP joints (*p* < 0.02) displaying dvOPs compared to those OA joints without dvOPs.

As reported before, we found central erosions in two DIP specimens and two PIP specimens and all displayed severe OA alterations [13]. Extending these findings we now report the presence of dvOPs in those four specimen. Regarding the GSS, the score amounted to 1 and 2 in DIP joints and was more pronounced in the PIP joints (GSS 3 in both PIP joints).

### Relationship of dorso-ventral osteophytes with patients’ age

**Fig. 4:**
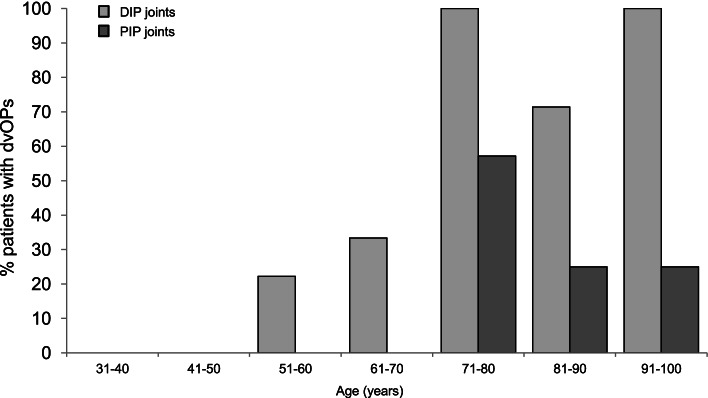
Prevalence of dorso-ventral osteophytes (dvOPs) in relation to patient age. dvOPs were identified on lateral view x-rays. Results are shown for 10-year age groups starting at the age of 31. DIP, distal interphalangeal joints; PIP, proximal interphalangeal joints

Patients’ ages ranged from 33 to 96 years (median 66 years). dvOPs could be detected in some patients between the age of 50 and 70 years; however, beyond the age of 70, the prevalence of dvOPs increased considerably (Fig. 4). As expected, age and the occurrence of dvOPs correlated well in DIP joints (*r*_s_ = 0.65; *p* <0.0001) but less strikingly in PIP joints (*r*_s_ = 0.32; *p* < 0.05). In joints displaying dvOPs, the patients’ age was significantly higher than in those that did not, both in DIP joints (joints with dvOPs: 77.4 ± 11.8 years vs joints without dvOPs: 56.2 ± 13.4 years; *p* < 0.0001) and PIP joints (joints with dvOPs: 79.7 ± 6.7 years vs joints without dvOPs: 65.4 ± 17.1 years; *p* < 0.04). Thus, the presence of dvOPs seems to be a function of increasing age and/or advanced OA.

No association of dvOPs with gender was found (data not shown).

## Discussion

Radiographic HOA is characterized by the presence of classic radiographic features, such as OP, JSN, subchondral sclerosis, subchondral cysts, or erosions [21–24]. The earliest changes found on x-ray images are OPs [25], which emerge in the course of the disease at sites of previous soft tissue alterations seen in earlier stages [23]. Interestingly, the most common site for OP development has been defined by a magnetic resonance imaging (MRI) study to be at the dorsal proximal side of the joint at the bone cartilage interface of the more proximal phalanx in both PIP and DIP joints [26]. Alterations at that location, however, cannot be captured by standard dp x-ray views due to the superimposition of other structures but could easily be seen on lateral/oblique view images as previously postulated [22, 27]. So far, however, almost no effort has been made to evaluate dorsally located OPs in HOA; this may be due to the fact that international societies do not regard additional radiographic views (besides standard dp images) as necessary in HOA diagnosis [7, 8] or that no radiographic score in HOA comprises the evaluation of oblique/lateral view images [15, 18, 28–33]. Nevertheless, especially in DIP and PIP joints, a predilection for dorsally based OP formation exists [21–23, 26], and its prevalence, as demonstrated in our previous study, is considerable [12].

Here, we provide a clear description of dorsally/ventrally located OPs and summarize these structures under dvOPs. Our description extends the common delineation of OPs defined as bony projections or bony outgrowth occurring most commonly at the margins of OA joints that are recognized radiographically most easily as bony excrescences at joint margins tangential to the x-ray beam [24]. We could show that dvOPs are quite common in DIP joints (but less common in PIP joints) compared to classic radiographic features of HOA. Nevertheless, the presence of dvOPs correlated well with histological (and radiographic) joint damage and was associated with synovial inflammation in both DIP and PIP joints. Interestingly, DIP and PIP-OA joints that did not display dvOPs showed less severe OA histological cartilage and radiographic damage, as well as less severe synovial inflammation. These findings suggest that the presence of dvOPs might be a marker of advanced HOA, as it reflects quite severe structural alteration/damage.

With regard to inflammatory changes, synovitis is generally recognized as a confounder in OA [34]. In HOA, mild synovitis is not uncommon [35], and synovitis was associated with severity of radiographic damage [34] and joint pain [35, 36]. However, prevalence data on synovitis detected by means of MRI or sonography are quite inconsistent and values range from 8 to 96.4% [37–40]. Our histology data show low synovitis in 40.5% of DIP and 33.3% of PIP joints as well as an association between synovial inflammation and joint damage and are thereby in line with the literature supporting the importance of synovial tissue in OA disease. Regarding OA pathogenesis, it is still unclear which tissue is the major player responsible for structural breakdown. Since histological cartilage damage correlates well with both bony changes and synovial inflammation, it is possible that activation of resident cells in the synovia contribute to cartilage destruction in the first place. However, blocking catabolically acting cytokines did not result in OA control [41–45]; thus, synovial inflammation could also be a reaction to other causes, such as mechanical strain.

We also evaluated the performance of radiographically detectable dvOPs with regard to diagnostic testing of HOA and report that dvOPs are highly specific (100%) and very sensitive particularly in DIP joints (81.82% for DIP and 54.55% for PIP joints). Classical radiographic features of HOA, such as lateral OPs and JSN, however, are generally sensitive (sensitivity 75–100%) but lack specificity (18–71%) [8]. Thus, the implementation of dvOPs evaluation could fill this gap, thereby improving the diagnostic value of plain radiographs in HOA. Nevertheless, for a quick evaluation regarding HOA in daily clinical practice, applying a standardized radiographic score on dp views is sufficient [12]; however, in some intricate cases, the inclusion of dvOPs could make a difference. Naturally, since this study was performed using a limited number of finger specimens, our findings might not accurately reflect HOA changes in the general population. Yet, our previous study on a HOA patient cohort with a comparable age range (40–85 years) supports our data, although prevalence of dvOPs in this cohort was lower [12]. Nonetheless, future longitudinal analyses could observe the evolution of dvOPs to determine the timepoint of initiation, as well as the speed of their development.

Besides the potential importance in the radiographic diagnosis of HOA, the detection of dvOPs could indicate function loss in HOA. dvOPs can, depending on their size, contribute to tendon damage and impairment of joint and hand function [46] and are associated with the occurrence of mucous cysts [47–49]. Intriguingly, these changes do not necessarily cause pain in the affected joints but may lead to progressive functional impairment [50] and ultimately to surgical excision of the cyst and the dvOP [46–48].

In this respect, the detection of dvOPs might not only be helpful during the diagnostic process of HOA but could also be an asset in identifying patients at risk for the development of tendon damage and worsening of joint function. This could be important insofar, as agents that modify structural changes in OA will be more intensively studied in the near future and eventually become available and such drugs may also be beneficial to patients who do not show significant levels of pain at a given point in time but in whom ongoing structural changes may lead to future symptoms and/or decline in joint function.

## Conclusion

In conclusion, dvOPs are quite prevalent in DIP joints and less prevalent in PIP joints. dvOPs correlate very well with structural joint damage, both on the histological and radiographic level, as well as with patients’ age. Moreover, especially in joints displaying dvOPs, synovial inflammation was present. Due to their good sensitivity and specificity, dvOPs could be a valuable additional tool in the diagnostic process and especially in research of HOA. As a matter of fact, for the evaluation of radiographic HOA in daily clinical practice the presence of dvOPs is not essential, but detecting the presence/absence of dvOPs can facilitate the diagnostic process and improve damage estimation. Possibly, the evaluation of dvOPs as markers of joint damage will find its way into radiographic atlases and will be implemented in radiographic scoring systems in the future.

## Data Availability

As parts of the obtained data are still processed, we cannot make the data set publicly available. However, we are happy to share our data individually upon specific request.

## Abbreviations

DIP: Distal interphalangeal
dp: Dorso/palmar
dvOP: Dorso-ventral osteophytes
HOA: Hand osteoarthritis
ICC: Intraclass correlation coefficient
K/L: Kellgren and Lawrence score
OA: Osteoarthritis
OP: Osteophyte
PIP: Proximal interphalangeal

## References

[1] Husic R, Finzel S, Stradner MH, Dreu M, Hofmeister A, Beham-Schmid C, Graninger WB, Fessler J, Dejaco C. Ultrasound in osteoarthritis of the hand: a comparison to computed tomography and histology. Rheumatology (Oxford). 2022;61(SI):SI73-SI80. 10.1093/rheumatology/keab526. 10.1093/rheumatology/keab526 34244721

[2] Lawrence RC, Felson DT, Helmick CG, Arnold LM, Choi H, Deyo RA (2008). Estimates of the prevalence of arthritis and other rheumatic conditions in the United States. Part II. Arthritis Rheum.

[3] Leung GJ, Rainsford KD, Kean WF (2014). Osteoarthritis of the hand I: aetiology and pathogenesis, risk factors, investigation and diagnosis. J Pharm Pharmacol.

[4] Reyes C, Garcia-Gil M, Elorza JM, Mendez-Boo L, Hermosilla E, Javaid MK (2015). Socio-economic status and the risk of developing hand, hip or knee osteoarthritis: a region-wide ecological study. Osteoarthr Cartil.

[5] Hochberg MC, Altman RD, April KT, Benkhalti M, Guyatt G, McGowan J (2012). American College of Rheumatology 2012 recommendations for the use of nonpharmacologic and pharmacologic therapies in osteoarthritis of the hand, hip, and knee. Arthritis Care Res.

[6] Mosher TJ, Walker EA, Petscavage-Thomas J, Guermazi A (2013). Osteoarthritis year 2013 in review: imaging. Osteoarthr Cartil.

[7] Hunter DJ, Arden N, Cicuttini F, Crema MD, Dardzinski B, Duryea J (2015). OARSI Clinical Trials Recommendations: hand imaging in clinical trials in osteoarthritis. Osteoarthr Cartil.

[8] Zhang W, Doherty M, Leeb BF, Alekseeva L, Arden NK, Bijlsma JW (2009). EULAR evidence-based recommendations for the diagnosis of hand osteoarthritis: report of a task force of ESCISIT. Ann Rheum Dis.

[9] Woodward AGF, Hedrick WR, Murphy C, Popovitch J, Martensen KM, Anthony BT, Steinsultz KL, Lampignano JPKL (2018). Terminology, positioning, and imaging principles. Bontrager’s Textbook of radiographic Positioning and Related Anatomy. 9th ed.

[10] Bhat AK, Kumar B, Acharya A (2011). Radiographic imaging of the wrist. Indian J Plast Surg.

[11] Schreibman KL, Freeland A, Gilula LA, Yin Y (1997). Imaging of the hand and wrist. Orthop Clin North Am.

[12] Staats K, Sunk IG, Weidekamm C, Kerschbaumer A, Becede M, Supp G (2020). Hand X-ray examination in two planes is not required for radiographic assessment of hand osteoarthritis. Ther Adv Musculoskelet Dis.

[13] Sunk IG, Amoyo-Minar L, Niederreiter B, Soleiman A, Kainberger F, Smolen JS, Bobacz K. Histopathological correlation supports the use of x-rays in the diagnosis of hand osteoarthritis. Ann Rheum Dis. 2013;72(4):572–7. 10.1136/annrheumdis-2011-200925. Epub 2012 May 12. 10.1136/annrheumdis-2011-200925 22580584

[14] Neame R, Zhang W, Deighton C, Doherty M, Doherty S, Lanyon P (2004). Distribution of radiographic osteoarthritis between the right and left hands, hips, and knees. Arthritis Rheum.

[15] Kellgren JH, Lawrence JS (1957). Radiological assessment of osteo-arthrosis. Ann Rheum Dis.

[16] Bijsterbosch J, Meulenbelt I, Watt I, Rosendaal FR, Huizinga TW, Kloppenburg M (2014). Clustering of hand osteoarthritis progression and its relationship to progression of osteoarthritis at the knee. Ann Rheum Dis.

[17] Bulstra SK, Buurman WA, Walenkamp GH, Van der Linden AJ (1989). Metabolic characteristics of in vitro cultured human chondrocytes in relation to the histopathologic grade of osteoarthritis. Clin Orthop Relat Res.

[18] Sunk IG, Amoyo-Minar L, Stamm T, Haider S, Niederreiter B, Supp G (2014). Interphalangeal Osteoarthritis Radiographic Simplified (iOARS) score: a radiographic method to detect osteoarthritis of the interphalangeal finger joints based on its histopathological alterations. Ann Rheum Dis.

[19] Krenn V, Morawietz L, Burmester GR, Kinne RW, Mueller-Ladner U, Muller B (2006). Synovitis score: discrimination between chronic low-grade and high-grade synovitis. Histopathology..

[20] Slansky E, Li J, Haupl T, Morawietz L, Krenn V, Pessler F (2010). Quantitative determination of the diagnostic accuracy of the synovitis score and its components. Histopathology..

[21] Feydy A, Pluot E, Guerini H, Drape JL (2009). Osteoarthritis of the wrist and hand, and spine. Radiol Clin N Am.

[22] Gupta KB, Duryea J, Weissman BN (2004). Radiographic evaluation of osteoarthritis. Radiol Clin N Am.

[23] Kaufmann RA, Logters TT, Verbruggen G, Windolf J, Goitz RJ (2010). Osteoarthritis of the distal interphalangeal joint. J Hand Surg [Am].

[24] Watt IDM, Brandt KD, DM, Lohmander LS (2003). Plain Radiographic features of osteoarthrits. Osteoarthritis. 2nd ed.

[25] Hutton CW, Higgs ER, Jackson PC, Watt I, Dieppe PA (1986). 99mTc HMDP bone scanning in generalised nodal osteoarthritis. I. Comparison of the standard radiograph and four hour bone scan image of the hand. Ann Rheum Dis.

[26] Tan AL, Grainger AJ, Tanner SF, Shelley DM, Pease C, Emery P (2005). High-resolution magnetic resonance imaging for the assessment of hand osteoarthritis. Arthritis Rheum.

[27] Allenspach P, Saupe N, Rufibach K, Schweizer A (2011). Radiological changes and signs of osteoarthritis in the fingers of male performance sport climbers. J Sports Med Phys Fitness.

[28] Altman RD, Gold GE (2007). Atlas of individual radiographic features in osteoarthritis, revised. Osteoarthr Cartil.

[29] Dougados M, Nguyen M, Mijiyawa M, Dropsy R (1990). Reproducibility of X-ray analysis of hand osteoarthrosis. Rhumatologie..

[30] Kallman DA, Wigley FM, Scott WW, Hochberg MC, Tobin JD (1989). New radiographic grading scales for osteoarthritis of the hand. Reliability for determining prevalence and progression. Arthritis Rheum.

[31] Kessler S, Dieppe P, Fuchs J, Sturmer T, Gunther KP (2000). Assessing the prevalence of hand osteoarthritis in epidemiological studies. The reliability of a radiological hand scale. Ann Rheum Dis.

[32] Verbruggen G, Veys EM (1995). Numerical scoring systems for the progression of osteoarthritis of the finger joints. Rev Rhum Engl Ed.

[33] Verbruggen G, Veys EM (1996). Numerical scoring systems for the anatomic evolution of osteoarthritis of the finger joints. Arthritis Rheum.

[34] Mathiessen A, Conaghan PG (2017). Synovitis in osteoarthritis: current understanding with therapeutic implications. Arthritis Res Ther.

[35] Haugen IK, Boyesen P, Slatkowsky-Christensen B, Sesseng S, van der Heijde D, Kvien TK (2012). Associations between MRI-defined synovitis, bone marrow lesions and structural features and measures of pain and physical function in hand osteoarthritis. Ann Rheum Dis.

[36] Fjellstad CM, Mathiessen A, Slatkowsky-Christensen B, Kvien TK, Hammer HB, Haugen IK (2020). Associations between ultrasound-detected synovitis, pain, and function in interphalangeal and thumb base osteoarthritis: data from the Nor-Hand cohort. Arthritis Care Res.

[37] Allado E, Wittoek R, Ferrero S, Albuisson E, Chary-Valckenaere I, Roux C (2020). Assessment of structural lesions, synovitis and bone marrow lesions in erosive hand osteoarthritis on MRI (0.3T) compared to the radiographic anatomical Verbruggen-Veys score. PLoS One.

[38] Damman W, Liu R, Bloem JL, Rosendaal FR, Reijnierse M, Kloppenburg M (2017). Bone marrow lesions and synovitis on MRI associate with radiographic progression after 2 years in hand osteoarthritis. Ann Rheum Dis.

[39] Kortekaas MC, Kwok WY, Reijnierse M, Watt I, Huizinga TW, Kloppenburg M (2010). Pain in hand osteoarthritis is associated with inflammation: the value of ultrasound. Ann Rheum Dis.

[40] Mathiessen A, Slatkowsky-Christensen B, Kvien TK, Hammer HB, Haugen IK (2016). Ultrasound-detected inflammation predicts radiographic progression in hand osteoarthritis after 5 years. Ann Rheum Dis.

[41] Aitken D, Laslett LL, Pan F, Haugen IK, Otahal P, Bellamy N (2018). A randomised double-blind placebo-controlled crossover trial of HUMira (adalimumab) for erosive hand OsteoaRthritis - the HUMOR trial. Osteoarthr Cartil.

[42] Chevalier X, Ravaud P, Maheu E, Baron G, Rialland A, Vergnaud P (2015). Adalimumab in patients with hand osteoarthritis refractory to analgesics and NSAIDs: a randomised, multicentre, double-blind, placebo-controlled trial. Ann Rheum Dis.

[43] Kloppenburg M, Peterfy C, Haugen IK, Kroon F, Chen S, Wang L (2019). Phase IIa, placebo-controlled, randomised study of lutikizumab, an anti-interleukin-1alpha and anti-interleukin-1beta dual variable domain immunoglobulin, in patients with erosive hand osteoarthritis. Ann Rheum Dis.

[44] Kloppenburg M, Ramonda R, Bobacz K, Kwok WY, Elewaut D, Huizinga TWJ (2018). Etanercept in patients with inflammatory hand osteoarthritis (EHOA): a multicentre, randomised, double-blind, placebo-controlled trial. Ann Rheum Dis.

[45] Verbruggen G, Wittoek R, Vander Cruyssen B, Elewaut D (2012). Tumour necrosis factor blockade for the treatment of erosive osteoarthritis of the interphalangeal finger joints: a double blind, randomised trial on structure modification. Ann Rheum Dis.

[46] Drapé JL CA, Bittoun J. Ungual and subungual disease. In: Guglielmi G, van Kuijk C, Genant HK, editors. Fundamentals of Hand and Wrist Imaging. 1st ed: Springer; 2001. p. 481-501.

[47] Kleinert HE, Kutz JE, Fishman JH, McCraw LH (1972). Etiology and treatment of the so-called mucous cyst of the finger. J Bone Joint Surg Am.

[48] Rizzo M, Beckenbaugh RD (2003). Treatment of mucous cysts of the fingers: review of 134 cases with minimum 2-year follow-up evaluation. J Hand Surg [Am].

[49] Waitayawinyu TTT, Trumble TE RG, Baratz M, Budoff JE, Slutsky DJ (2016). Ganglion, Mucous cyst, and Carpal Boss. Principles of Hand Surgery and Therapy. 3rd ed.

[50] Merle MVL, Merle MRS (2009). Arthrose der Fingergelenke. Chirurgie der Hand: Rheuma, Arthrose, Nervenengpässe.

